# Cypermethrin-Induced Toxic Effect on Glycogen Metabolism in Estuarine Clam, *Marcia Opima* (Gmelin, 1791) of Ratnagiri Coast, Maharashtra

**DOI:** 10.1155/2012/576804

**Published:** 2012-04-17

**Authors:** Medha Tendulkar, Arvind Kulkarni

**Affiliations:** Department of Zoology, R.P. Gogate College of Arts and Science and R.V. Jogalekar College of Commerce, Maharashtra, Ratnagiri 415612, India

## Abstract

Cypermethrin is a synthetic pyrethroid class of insecticide. Toxic effects of cypermethrin were studied by selecting *Marcia opima* as an animal model. Cypermethrins effect on the total glycogen content of mantle, gill, foot, hepatopancreas, male gonad and a female gonad of an estuarine clam, *Marcia opima* was examined. The clams were exposed to 1.58 ppm cypermethrin for acute and 1/10th of that concentration for chronic treatment. It was found that there was a decrease in glycogen content in various tissues as compared to control. In LC_0_ and LC_50_ groups, glycogen was decreased in all tissues except in hepatopancreas compared to control. This decrease is greater in mantle, gill, and foot in LC_50_ group than the decrease in those tissues of LC_0_ group. In chronic exposure it was found that glycogen was decreased in mantle, foot, male gonad, and female gonad when compared to the control group except in gill and hepatopancreas. Decrease in glycogen content indicates greater utilization of glycogen for metabolic purposes and too combat with cypermethrin stress. The significant increase in glycogen content in gill and hepatopancreas may be a reaction to the increase in energy demand.

## 1. Introduction

To meet the ever-increasing demand of the rising human population, there has been surging increase in the use of agricultural chemicals like pesticides to preserve the standing crops from the attack of pests and to boost crop production. Due to injudicious and indiscriminate use of pesticides, the natural water resources such as lakes, reservoirs, rivers, ponds, paddy-fields, streams, and other low-lying areas are getting polluted all over the world. Pesticides, affect the whole ecosystem, particularly the aquatic ones, leading to unwarranted mortality of aquatic biota, in general and fish in particular as revealed by several workers [1–7].

 Pesticides and herbicides have created two major problems by persisting and accumulating in the environment, therefore, contaminating numerous plants and animals, and secondly they affect human health directly or indirectly. Pesticides are air, water, and soil pollutants and can have harmful effects on plants and human beings. They can also be hazardous for all forms of aquatic life. Most pesticides such as DDT, DDE, DDD, dieldrin, heptachlor epoxide, and cypermethrin, and most herbicides such as 2,3,5_T(2,4,5 trichlorophenoxyacetic acid) dioxin have been extensively used for disease control on crop destroying insects.

 Cypermethrin (C_22_H_19_Cl_2_NO_3_) is a synthetic pyrethroid class of insecticide. It is commonly used to control various pests, including moth pests of cotton, fruit, and vegetable crops [8]. It is also used for crack, crevice, and spot treatment to control insect pests in stores, warehouses, industrial buildings, houses and apartments, greenhouses, laboratories, ships, rail-cars, buses, trucks, and aircrafts. It may also be used in nonfood areas in schools, nursing homes, hospitals, restaurants, hotels, and food processing plants [9]. It is being used in veterinary practice against ectoparasites. On the market, it is available in form like Cymbush EC, Cynoff EC, Cynoff WP, Demon EC, and Demon WP pesticide concentrates. In Ratnagiri, it is mainly used against mango hoppers, mango mealy bugs, aphids, and other insect pests of mango. Due to this, Cypermethrin is getting concentrated in the aquatic bodies like rivers and estuaries.

 Clams are abundant along the coast of Ratnagiri (Maharashtra) and are important because they are commonly used as food. Shells are mainly used as a raw material for lime factories along the coast. In spite of this fact, miniscule attention has been paid by researchers in regards to the effect of pollution on estuarine clams. Clams are known to be tolerant to pesticides accumulation and have a relatively long life span. Since biochemical assessment is a useful tool for measuring environmental quality, the present work is aimed to study the effect of Cypermethrin on glycogen metabolism in estuarine clam, *Marcia opim, *(Gmelin, 1791). 

## 2. Materials and Methods

The experimental clams, *Marcia opima *(Gmelin) used for the present study were collected from Bhatye estuarine region, of Ratnagiri coast, in Maharashtra state. The clams of medium size measuring 3.5–4 cms in length and weighing 16–20 grams were selected, brought to the laboratory and stocked in the plastic containers containing filtered, aerated estuarine water, for 48 hours. During this period water was changed three times a day. While studying the toxicity, no food was given to the animals before or during the experimental period. The clams that acclimatized well to the laboratory condition were grouped in 10 and kept in plastic containers containing 5 liters of filtered estuarine water. Static bioassay tests were conducted for 96 hours (acute) and 30 days (chronic) by using cypermethrin (25% EC). For every experiment, a control group of 10 clams was also run simultaneously. All the experiments were conducted in natural day light rhythm. The temperature, pH, dissolved oxygen, and salinity of the water used to hold the clams were recorded during each experiment.

The toxicity tests were repeated for three times, and LC_0_ and LC_50_ values were determined. All the experiments were carried out on freshly collected clams in Summer season (April/May). After finding the LC_50_ dose, the clams are exposed to 1/10th dose of cypermethrin for 30 days (chronic). After studying the 96 h (acute) and 30 days (chronic) toxicity of Cypermethrin to *Marcia opima* various tissues (gills, mantle, hepatopancreas, foot, male, and female gonad) of the control, LC_0_ and LC_50_ groups from acute exposure and chronic exposure were pooled, weighed, and dried in an oven at 70°C until a constant weight was obtained. Tissues were powdered, and oven-dried tissue powder was analyzed for total glycogen [10]. The data obtained was statistically analyzed for confirmation of results by using student's *t*-test.

## 3. Results and Discussions

### 3.1. Acute Exposure

Alterations in glycogen content after acute exposure to cypermethrin are shown in Table 1. During Summer, glycogen content with in the control group was present in ascending order of gill < hepatopancreas < male gonad < foot < mantle < female gonad. The glycogen content was 11.858 ± 0.864, 14.666 ± 0.174, 19.85 ± 0.402, 20.9 ± 0.408, 23.575 ± 0.198, and 39.6 ± 0.454 mg/100 mg dry tissue in respective organs. In LC_0_ group (1.11 ppm), the glycogen content was present in ascending order of gill < male gonad < foot < hepatopancreas < mantle < female gonad, with 9.31 ± 0.377, 17.235 ± 0.119, 18.524 ± 0.275, 19.23 ± 0.589, 20.525 ± 0.119, and 30.52 ± 0.286 mg/100 mg dry tissue, respectively. When compared to the control group, there was 31.11% significant increase of the glycogen content in hepatopancreas and decrease in other organs. In LC_50_ group (1.58 ppm), the glycogen content occurred in ascending order of gill < foot < male gonad < mantle < hepatopancreas < female gonad with 7.486 ± 0.117, 14.589 ± 0.289, 16.11 ± 0.197, 16.175 ± 0.126, 21.786 ± 0.150, and 28.52 ± 0.091 mg/100 mg dry tissue in respective organs. As compared to control group, there was a significant decrease of the glycogen content in all tissues except hepatopancreas.

### 3.2. Chronic Exposure

Alterations in glycogen content after chronic exposure to cypermethrin are shown in Table 2. The control group showed glycogen content in ascending order of gill < hepatopancreas < male gonad < foot < mantle < female gonad with 11.456 ± 0.178, 15.508 ± 0.129, 19.613 ± 0.111, 20.833 ± 0.067, 23.465 ± 0.167, and 39.182 ± 0.128 mg/100 mg dry tissue, respectively. In chronic group, glycogen content was present in ascending order of foot < male gonad < gill < mantle < hepatopancreas < female gonad with 15.526 ± 0.087, 17.264 ± 0.056, 19.581 ± 0.045, 20.857 ± 0.053, 21.244 ± 0.102, and 32.619 ± 0.062 mg/100 mg dry tissue in respective organs. As compared to the control group, there was a significant increase in gill and hepatopancreas and significant decrease in all other tissues.

 The studies on biochemical changes make it possible to define the dose response relationship, threshold limit value, and reversible and irreversible nature of pollutant effect. In addition, the biochemical indices of toxicity derived after a relatively short exposure time may be useful in predicting the appropriate threshold concentration for the development of chronic effects [11].

 In estuarine clams, glycogen is the prime source of energy for carrying out various life activities but due to the pesticide stress, the prime energy source is affected severely, and it was a negative effect on various processes in the clam's body [12]. In LC_0_ and LC_50_ groups, Glycogen content was decreased in gill, mantle, foot, male gonad, and female gonad, hepatopancreas being the exception because these organs are more active, and they require large amount of energy. This energy demand is solved by utilizing reserve food material in the form of glycogen. In chronic group, there was a significant decrease in all tissues except for the gill and hepatopancreas, respectively. Decline in glycogen in various tissues may be due to stress resulting in breakdown of tissue glycogen [13] to meet the energy demand under toxic stress of pesticide. Decrease in glycogen content indicates greater utilization of glycogen for metabolic purposes and to combat with Cypermethrin stress. The significant increase in glycogen content in hepatopancreas may be due to the increased energy demand. While studying cadmium impact on estuarine clams like *Katelysia opima*, *Meretrix meretrix,* and* Paphia laterisulca*, Kumbhar [14] showed similar type of results.

 Metabolic activity of the clams showed utilization of the biochemical energy to counteract the toxic stress. After acute exposure of Cypermethrin, clams showed remarkable changes in the biochemical composition of the glycogen content of the various tissues like gills, foot, mantle, hepatopancreas, male gonad, and female gonad. In general, there was decrease in glycogen level in LC_0_ and LC_50_ groups in mantle, gill, foot, male gonad, and female gonad, hepatopancreas being the exception. The clams from LC_50_ group showed significant decrease in glycogen content as compared to LC_0_ group. In the present study, the elevation of glycogen content in gills of LC_0_ group might be accounted for increased lipolysis for copping up with increased glycogen demand. 

## Figures and Tables

**Table 1:** Cypermethrin induced alterations in the total glycogen content of *K. opima* after acute exposure. (Results expressed in mg/100 mg dry wt. basis).

Tissue	Control (mg/100 mg)	LC_0_ Group (mg/100 mg)	LC_50_ Group (mg/100 mg)
Mantle	23.575 ± 0.198	20.525 ± 0.119 (−12.93)***	16.175 ± 0.126 (−31.38)***
Gill	11.858 ± 0.864	9.31 ± 0.377 (−21.48)***	7.486 ± 0.117 (−36.86)***
Foot	20.9 ± 0.408	18.524 ± 0.275 (−12.82)***	14.589 ± 0.289 (−30.19)***
Hepatopancreas	14.666 ± 0.174	19.23 ± 0.589 (31.119)***	21.786 ± 0.150 (48.5)***
Male gonad	19.85 ± 0.402	17.235 ± 0.119 (−13.17)***	16.11 ± 0.197 (−18.84)***
Female gonad	39.6 ± 0.454	30.52 ± 0.286 (−22.92)***	28.52 ± 0.091 (−27.97)***

Values in parenthesis are percentage difference.

SD of five readings ***P < 0.001.

**Table 2:** Cypermethrin induced alterations in the total glycogen content of *K. opima* after chronic exposure. (Results expressed in mg/100 mg dry wt. basis).

Tissue	Control (mg/100 mg)	Chronic Group (mg/100 mg)
Mantle	23.465 ± 0.167	20.857 ± 0.053 (−11.11)***
Gill	11.456 ± 0.178	19.581 ± 0.045 (70.92)***
Foot	20.833 ± 0.067	15.526 ± 0.087 (−25.47)***
Hepatopancreas	15.508 ± 0.129	21.244 ± 0.102 (36.99)***
Male gonad	19.613 ± 0.111	17.264 ± 0.056 (−11.97)***
Female gonad	39.182 ± 0.128	32.619 ± 0.062 (−16.74)***

Values in parenthesis are percentage difference.

SD of five readings ***P < 0.001.
